# Annexin A6 in Eye-Lavage Extracellular Vesicles—a Candidate Biomarker for Sex-Specific Differences in Mild Hypoxic–Ischaemic Brain Damage—Findings from an Explorative Neonatal Mouse Model Study

**DOI:** 10.3390/ijms27198875

**Published:** 2026-10-05

**Authors:** Sigrun Lange, Sarah R. Needham, Igor Kraev, Benjamin M. Davis, Mariya Hristova

**Affiliations:** 1 Pathobiology and Extracellular Vesicles Research Group, School of Life Sciences, University of Westminster, London W1W 6UW, UK; 2 Department of Neonatology, EGA Institute for Women’s Health, University College London, London WC1E 6BT, UK; 3 Lasers for Science Facility, Central Laser Facility, UKRI: Science & Technology Facilities Council, Rutherford Appleton Laboratory, Oxfordshire OX11 0QX, UK; sarah.needham@stfc.ac.uk (S.R.N.); benjamin.davis@stfc.ac.uk (B.M.D.); 4 Electron Microscopy Suite, Faculty of Science, Technology, Engineering and Mathematics, Open University, Milton Keynes MK7 6AA, UK; igor.kraev@open.ac.uk

**Keywords:** annexin A6 (ANXA6), brain, eye lavage, extracellular vesicles (EVs), hypoxic ischaemic injury (HI), neonate, sex differences, time window

## Abstract

Extracellular vesicles (EVs) play important roles in cell communication, including via transfer of cargo proteins, and are promising biomarkers for pathobiological processes. Reliable biomarkers for mild neonatal hypoxia ischaemia (HI) are scarce. Wrongly determined HI severity often leads to adverse long-term neurological outcomes, with more detrimental effects in males. Therefore, identification of sex-specific markers for HI is of great interest. We recently reported the potential of EVs from eye lavage as novel non-invasive biomarkers of neonatal HI brain injury, differing in proteomic cargoes between mild and severe HI brain insults. In continuation, the current study therefore focused on assessing sex-specific differences in the same mouse model of mild HI with respect to Annexin A6 (ANXA6) EV protein cargo, at different time points (0 h, 24 h and 48 h) post HI insult. Using direct stochastic optical reconstruction microscopy (dSTORM) quantifying and co-localising ANXA6 to EV subtypes, our results indicate more marked changes in males compared with females, with a peak of positive ANXA6 detection in EVs at 24 h post HI in males. PanEV+TT+ EVs contained most ANXA6, followed by the PanEV+-labelled EVs. In the females, less ANXA6-positive staining was observed in EVs of all three time points in the HI groups compared with the males. In the sham and naïve control animals, ANXA6 detection suggested developmental reduction of ANXA6 in both sexes. However, in the male HI groups that developmental reduction was eliminated and ANXA6 remained at an increased level at all three time points. In addition, EV subtype labelling showed some differences between time points within each sex group and between sexes. PanEV+ single labelled EVs were more abundant in the females compared with the males in the 0 h and 24 h post-HI groups and PanEV+TT+-labelled EVs were more abundant in females than males at the 0 h post-HI time point. Our pilot findings indicate that increased ANXA6 labelling in eye-lavage EVs is associated with the mild HI model with more marked changes in males, where ANXA6 positivity peaked at 24 h post HI. Outcomes of this study contribute to EV-based biomarker development for HI diagnostics, with potential clinical value, including sex-specific differences. Future studies should include validation in further biological replicates in larger cohort sizes and assessment in human neonatal clinical samples.

## 1. Introduction

Neonatal hypoxia ischaemia (HI) affects 1–3 per 1000 live births and is a major cause of infant mortality and morbidity. HI leads to adverse long-term neurological outcomes, including mental and motor abnormalities, behavioural deficits, developmental delay, learning disabilities, and cerebral palsy [1,2,3]. In addition, HI can contribute to cortical visual impairment (CVI) and retinopathy of prematurity, which in its mild form usually heals on its own within four months, but severe cases require treatment to prevent complications such as vision loss and retinal detachment [4,5,6]. The eye tissue forms directly from the brain during embryonic development, and both the retina and optic nerve are classified as part of the central nervous system. Therefore, it has been suggested that the eye can reflect damage occurring to the brain and may offer an approach for detecting specific time windows for early brain-related changes [7]. Limited knowledge is currently available regarding the response of the eye to HI damage, including in neonates, and whether the eyes’ cellular activation reflects the processes observed in the brain. Therefore, it will be of great value to develop new methods, focussing on characterising extracellular vesicle (EV) signatures from eye lavage as robust biomarkers for brain and eye pathologies [8].

EVs are released from cells and play key roles in cellular communication through transfer of diverse cargoes, comprising lipids, RNA species, DNA and proteins. EVs can be isolated from most body fluids and are valuable biomarkers with clinical relevance [9,10]. Their use as non-invasive biomarkers for brain-related injury, including for neonatal HI, is of considerable interest [8,11]. The use of EVs as biomarkers for monitoring the downstream effects of HI is understudied to date. Our recent work using eye-lavage EVs to gain insights into HI-related damage highlights their potential as novel non-invasive biomarkers [8]. Using EVs from eye lavage in neonatal HI offers a different approach from EVs isolated from tears, as in neonates tear production is limited. Hence, approaches used to assess EVs in tears of various ocular diseases [12,13,14,15,16,17] would not be feasible in neonates.

In neonatal HI the only clinically approved treatment is therapeutic hypothermia (TH), which reduces development of cognitive impairments and incapacities but does not guarantee total recovery from neonatal HI and 40% of treated infants still develop disabilities [18]. TH is a proven intervention for moderate/severe HI but has been largely excluded for mild cases, where it can cause adverse effects [19,20]. Therefore, new biomarkers are urgently needed for assessment of HI severity and damage. Furthermore, sexual dimorphism observed in HI points to more severe outcomes in males, including more pronounced neurological deficits, compared with females [21,22]. Based on our recent findings in a mouse model of neonatal HI, where we established a new protocol for eye-lavage EVs and explored their proteomic content in mixed sex groups of mild and severe HI [8], we suggest that eye-lavage EVs provide a valuable tool to assess brain injury in neonatal HI, including sex-specific differences. Our previous research identified some profile changes in eye-lavage EV proteomic cargoes in both mild and severe HI at 48 h post injury, in mixed sex groups, warranting further investigations of selected EV-associated protein targets and assessment of different time points post HI, alongside putative sex-specific differences. Therefore, our current study focused on further assessment of Annexin A6 (ANXA6) in mild HI, which we identified as a cargo protein marker in eye-lavage EVs of a mild 30 min HI mouse model in a mixed sex group at 48 h post injury [8]. ANXA6 is a calcium-dependent membrane-binding protein which binds to damaged cell membranes and has protective roles in neuronal injury and in neuronal membrane dynamics [23,24]. ANXA6 affects proinflammatory cytokine secretion as well as EV release [25]. In a gene expression profiling study, ANXA6 was identified as a neuroinflammatory marker linking neonatal HI brain injury to Alzheimer’s disease [26]. In a mouse model of middle cerebral artery occlusion ANXA6 enhanced synaptic plasticity and protected against transient cerebral ischaemia [27]. Given the links to neuronal injury, including in HI, we hypothesise that ANXA6 shows different levels in EV cargoes of male versus female eye-lavage EVs at different time points (0 h, 24 h and 48 h) post mild HI insult.

## 2. Results

### 2.1. EV Characterisation and EV Analysis by Nanoparticle Tracking Analysis (NTA)

**Figure 1:**
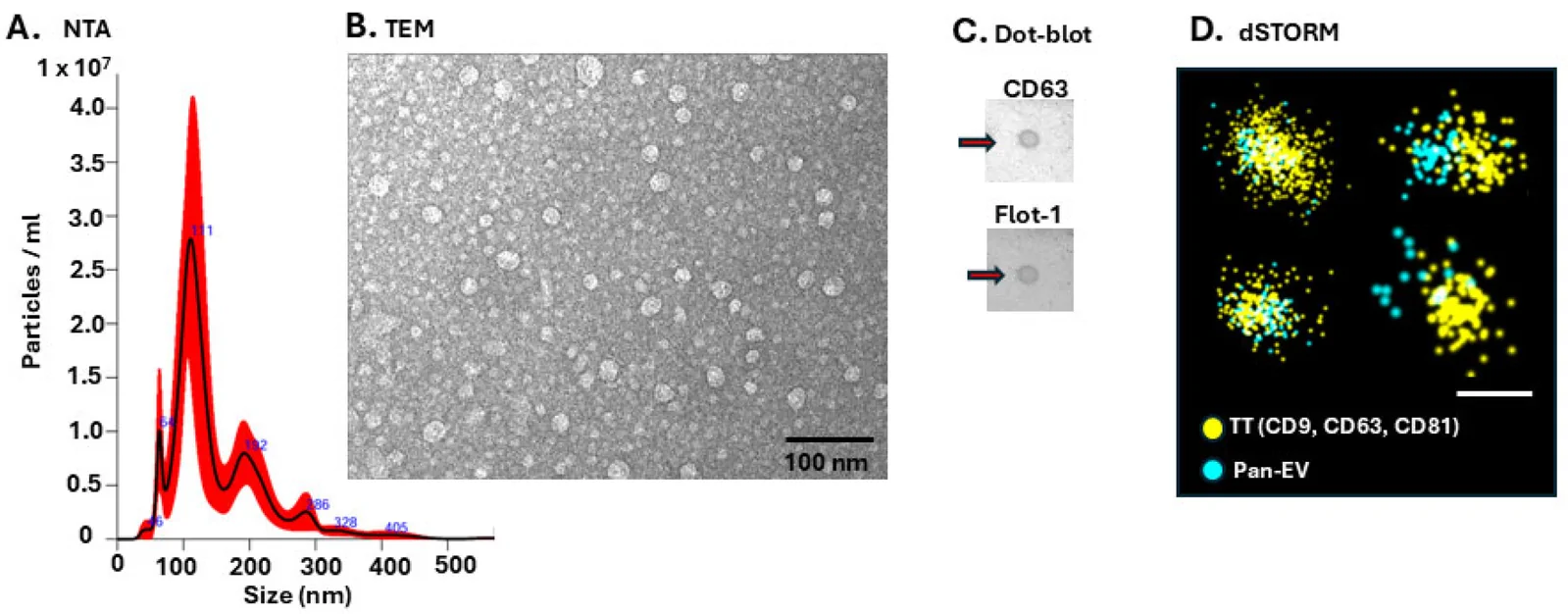
Characterisation of eye-lavage EVs. (**A**) NTA exemplary size distribution curve, showing typical profiles in approximately 50–400 nm size range. (**B**) TEM imaging of eye-lavage EVs (scale bar = 100 nm). (**C**) Dot blot of eye-lavage EVs, showing positive for CD63 and Flotillin-1 (Flot-1) as highlighted by the arrows. (**D**) Imaging of EVs by dSTORM for tetraspanin trio (TT, yellow) and PanEV (blue) markers, scale bar = 100 nm for all images.

Eye-lavage EVs were characterised by nanoparticle tracking analysis (NTA) for quantification and size distribution (Figure 1A; Supplementary Figure S1), by transmission electron microscopy (TEM) imaging (Figure 1B), dot blotting for CD63 and Flotillin-1 (Figure 1C) and by direct stochastic optical reconstruction microscopy (dSTORM) imaging for tetraspanin trio (TT—CD9, CD63, CD81; yellow) and PanEV (blue) markers (Figure 1D) using the ONI EV2 profiler kit.

**Figure 2:**
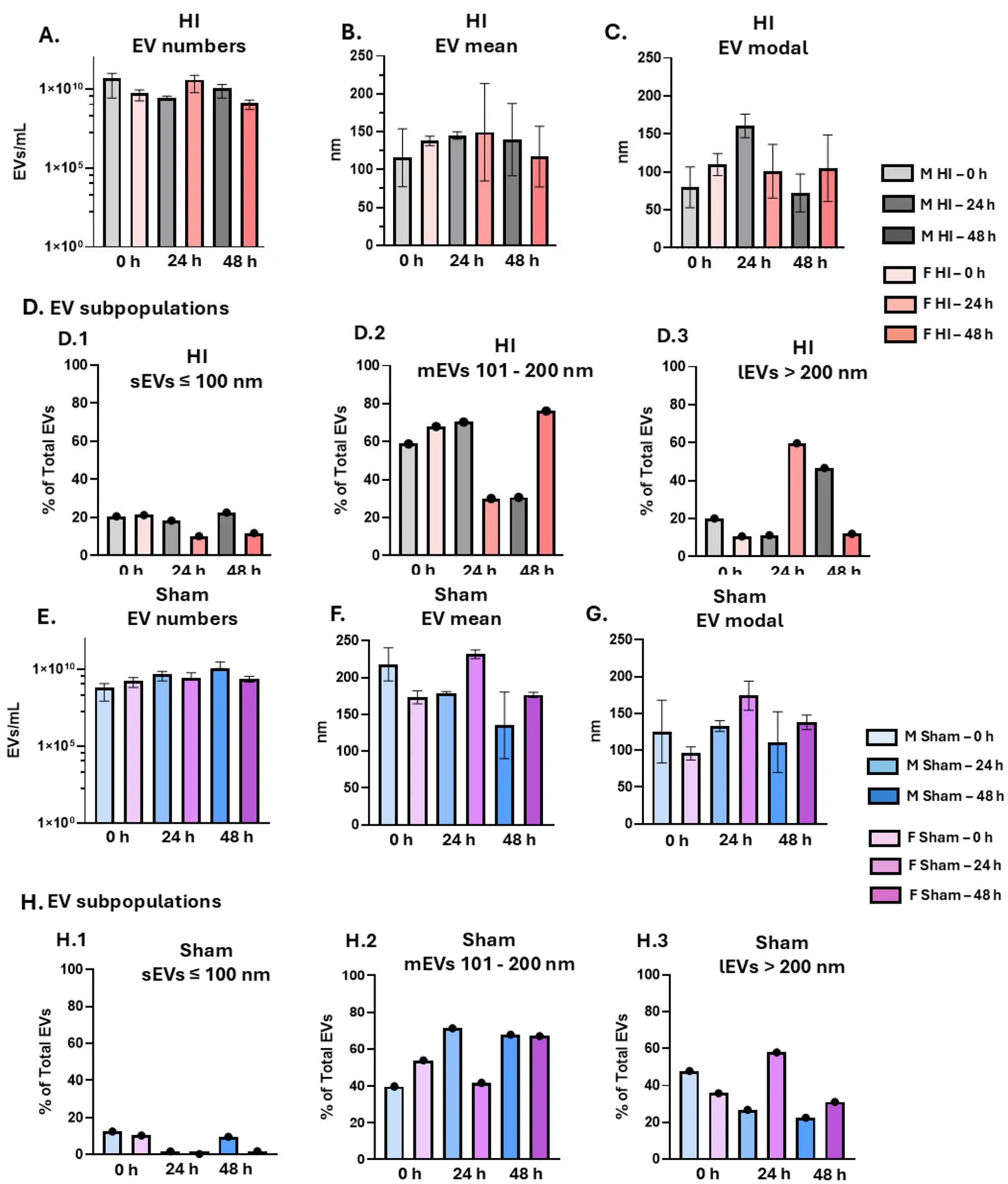

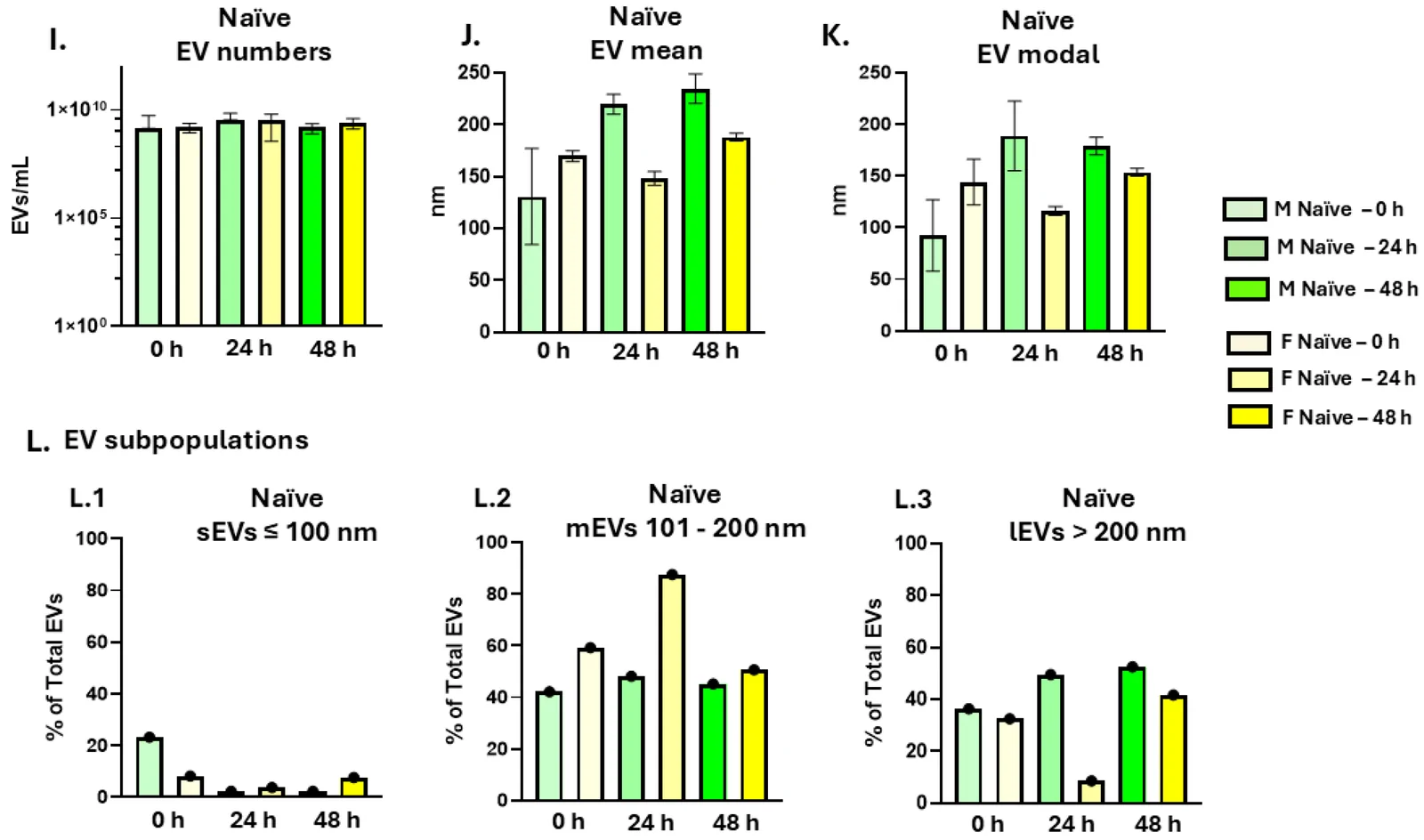
Nanoparticle tracking analysis (NTA) of EV distribution in males and females, at 0 h, 24 h and 48 h post 30 min HI (**A**–**D**) and in sham (**E**–**H**) and naïve (**I**–**L**) control groups for the same time points. (**A)** EV numbers of HI groups—mean with error bars (SEM) representing five repetitive reads from a pool of n = 10 eyes; (**B**) EV mean size of HI groups—mean with error bars representing five repetitive reads from a pool of n = 10 eyes; (**C)** EV modal size of HI groups—mean with error bars representing five repetitive reads from a pool of n = 10 eyes; (**D**) EV subpopulation distribution of HI groups showing small EVs (sEVs, 0–100 nm, **D.1**), medium EVs (mEVs, 101–200 nm, **D.2**) and large EVs (lEVs, > 200 nm, **D.3**) as a proportion of total EV percentage per group. (**E**–**H**) Sham group EV analysis from NTA readings for EV numbers (**E**), EV mean size (**F**), EV modal size (**G**), EV subpopulation distribution (**H.1**–**H.3**). (**I**–**L**) Naïve group EV analysis from NTA readings for EV numbers (**I**), EV mean size (**J**), EV modal size (**K**), EV subpopulation distribution (**L.1**–**L.3**). All graphs present results from single pool data per group (n = 10 eyes) with 5 technical replications. The NTA curves per each pooled group (n = 10 eyes per group) are shown in Supplementary Figure S1.

For all groups, EVs were quantified (EVs/mL) per sample group at 0 h, 24 h and 48 h post HI, as well as in naïve and sham control groups, at all three time points, for males and females respectively, showing similar EV yield in eye lavage from all groups (Figure 2A,E,I). EV mean sizes were assessed in all groups, with some variation observed between groups and smaller EV profiles in the HI groups (Figure 2B,F,J). EV modal size showed some shift to larger EVs in the 24 h post-HI male group, as well as female shams at 24 h, and naïve males at the 24 h time point, (Figure 2C,G,K). Given that the NTA was based on pools of n = 10 eyes per group, with five repetitive reads merged for each analysis, no further statistical comparison was carried out for this dataset. Additional assessment on EV subpopulations based on size estimation by NTA (estimated as a % of total EVs; Figure 2D,H,L) indicated some changes in small EVs (sEVs ≤ 100 nm) with higher levels in HI males than HI females at 24 h and 48 h (Figure 2D.1). For medium EVs (mEVs 101–200 nm) higher levels were observed in the HI males at 24 h compared with HI females, while in the HI females, levels were higher at 48 h compared with HI males (Figure 2D.2). Large EVs (lEVs > 200 nm) were at higher levels in HI females at 24 h post HI but at 48 h post HI in the males (Figure 2D.3).

When comparing the three time points post HI within each sex group, no change was observed in sEVs in the males between time points, while in the females there were lower levels of sEVs observed at 24 h and 48 h post HI compared with the 0 h group (Figure 2D.1). Lower levels of mEVs were observed in the males at 48 h compared with 0 h and 24 h post-HI groups; while in the females levels of mEVs were lower at the 24 h time point compared with 0 h and 48 h groups (Figure 2D.2). When comparing the lEVs, these were increased in males at 48 h post HI compared with 0 h and 24 h post HI and in the females at 24 h compared with 0 h and 48 h post HI (Figure 2D.3).

In the sham groups some changes in EV subpopulations were observed, with a reduction of sEVs in males and females at 24 h, and the females in the 48 h group, compared with the 0 h groups (Figure 2H.1). Medium EVs were higher in the 0 h female group, but lower in females in the 24 h group, compared with the males (Figure 2H.2). Large EVs were higher in the females at 24 h compared with the males (Figure 2H.3). In the naïve animals, sEVs were comparatively lower at the 24 h (males and females) and 48 h time points (males) compared with the 0 h groups (Figure 2L.1), while mEVs were highest in the female 24 h group, which correlated to low detection of large EVs in the female 24 h group (Figure 2L.3).

### 2.2. EV Surface Marker Detection by dSTORM

**Figure 3:**
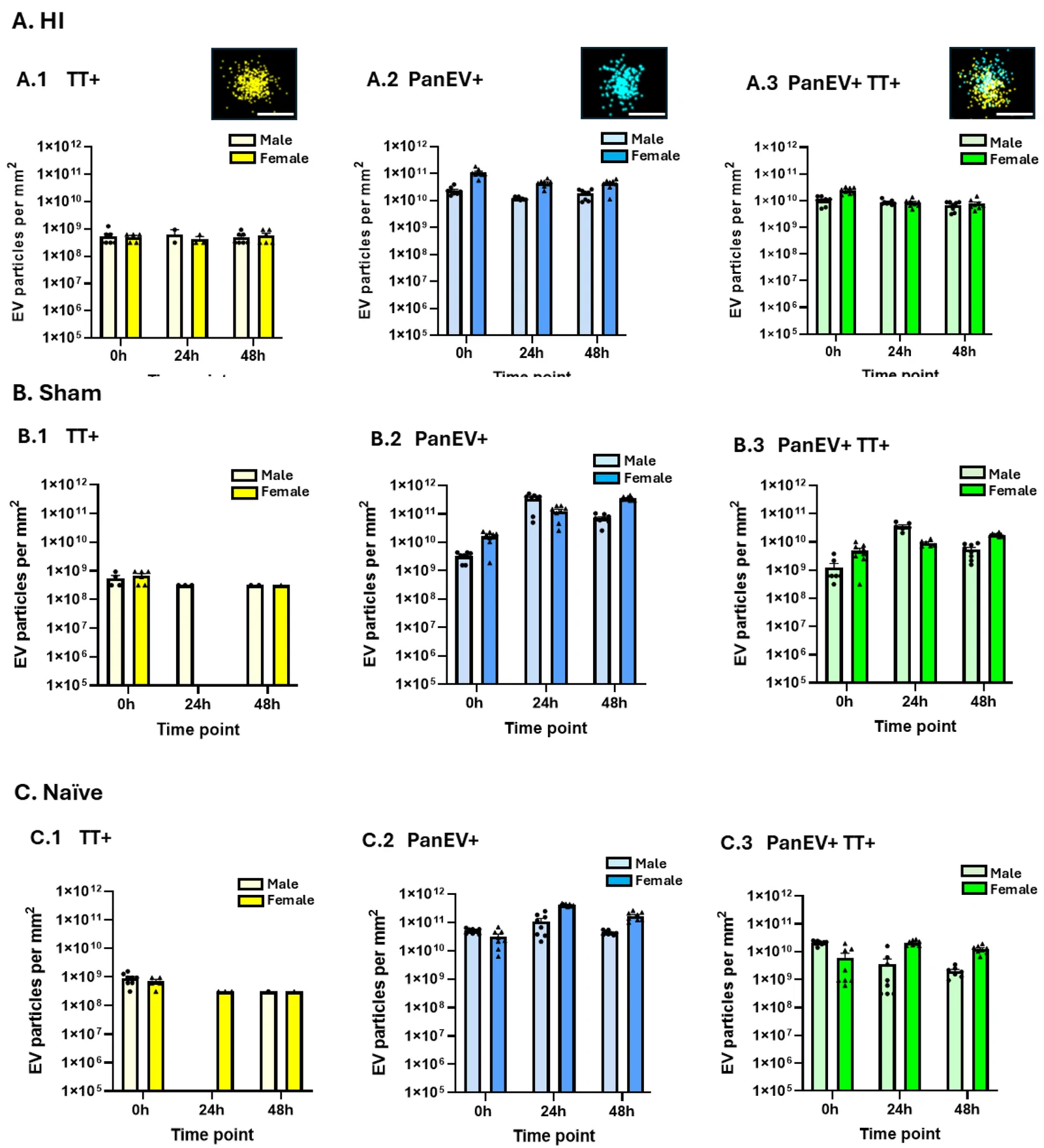
EVs labelled by dSTORM for EV specific surface markers, across all groups. (**A**) HI groups: (**A.1**) Only TT+-labelled EVs; (**A.2**) Only PanEV+-labelled EVs; (**A.3**) Co-labelled PanEV+ and TT+ EVs. (**B**) Sham groups: (**B.1**) Only TT+-labelled EVs; (**B.2**) Only PanEV+-labelled EVs; (**B.3**) Co-labelled PanEV+ and TT+ EVs. (**C**) Naïve groups: (**C.1**) Only TT+-labelled EVs; (**C.2**) Only PanEV+-labelled EVs; (**C.3**) Co-labelled PanEV+ and TT+ EVs.; positive readings taken from ONI chips in 6–12 fields of view per group; *y*-axis represents positively labelled EV particles per mm^2^.

Analysis of positively labelled EVs by dSTORM imaging confirmed a mixture of PanEV- and TT-positive EVs across all groups and positive EV staining in all groups was compared for TT-positive labelled EVs (Figure 3A.1,B.1,C.1), PanEV-positive labelled EVs (Figure 3A.2,B.2,C.2) and EVs co-labelled for PanEV and TT markers (Figure 3A.3,B.3,C.3). Overall, TT+ single-stained EVs (Figure 3A.1,B.1,C.1) were observed at a lower proportion than PanEV+ single-stained EVs (Figure 3A.2,B.2,C.2) and PanEV-TT+ co-stained EVs (Figure 3A.3,B.3,C.3) in all groups, which were associated with the proportional detection of EV subpopulations in the NTA for all groups. When assessing sex-specific differences and differences between time points, no alterations were observed for the TT+ single-labelled EVs for the HI groups (Figure 3A.1), while in the sham female 24 h group detection was negligible (Figure 3B.1), as well as in the male 24 h naïve group (Figure 3C.1). The PanEV+ single-labelled EVs were observed at a higher proportion in the females than in the males at all three time points in the HI groups (Figure 3A.2). In the sham animals, an observed proportional increase in PanEV+ EVs was detected in both sexes at the 24 h and 48 h time points, compared with 0 h groups, and levels were higher in the males at 24 h than in females but lower than females at the 48 h time point (Figure 3B.2). In the naïve animals, PanEV+ detection was higher in females than in males at the 24 h and 48 h time points (Figure 3C.2). For the PanEV+TT+ co-labelled EVs, numbers of positive EVs were observed to be proportionally higher in the females than in males at 0 h post HI and proportionally lower in the 24 h and 48 h post-HI females compared with the 0 h female group (Figure 3A.3); no such differences were observed between 0 h, 24 h and 48 h in the male HI groups. In the sham animals, PanEV+TT+ co-labelled EVs were observed to be proportionally increased in males at 24 h and in the females at 48 h compared with 0 h controls (Figure 3B.3). In the naïve animals, PanEV+TT+ co-labelled EVs were proportionally higher in males at 0 h but proportionally lower in males when compared with females at 24 h and 48 h (Figure 3.C.3). PanEV+TT+ staining was observed to be proportionally increased in females at 24 h and 48 h compared with 0 h, while in males it appeared proportionally lower at 24 h and 48 h than in the 0 h group. Due to the nature of the datasets, using a pool of n = 10 with the analysis carried out in 6–12 representative fields of view per sample group, further in-depth statistical analysis was not carried out, and the data interpretation therefore remains explorative and descriptive.

### 2.3. Annexin A6 EV Cargo Analysis and Co-Localisation to EV Subtypes

**Figure 4:**
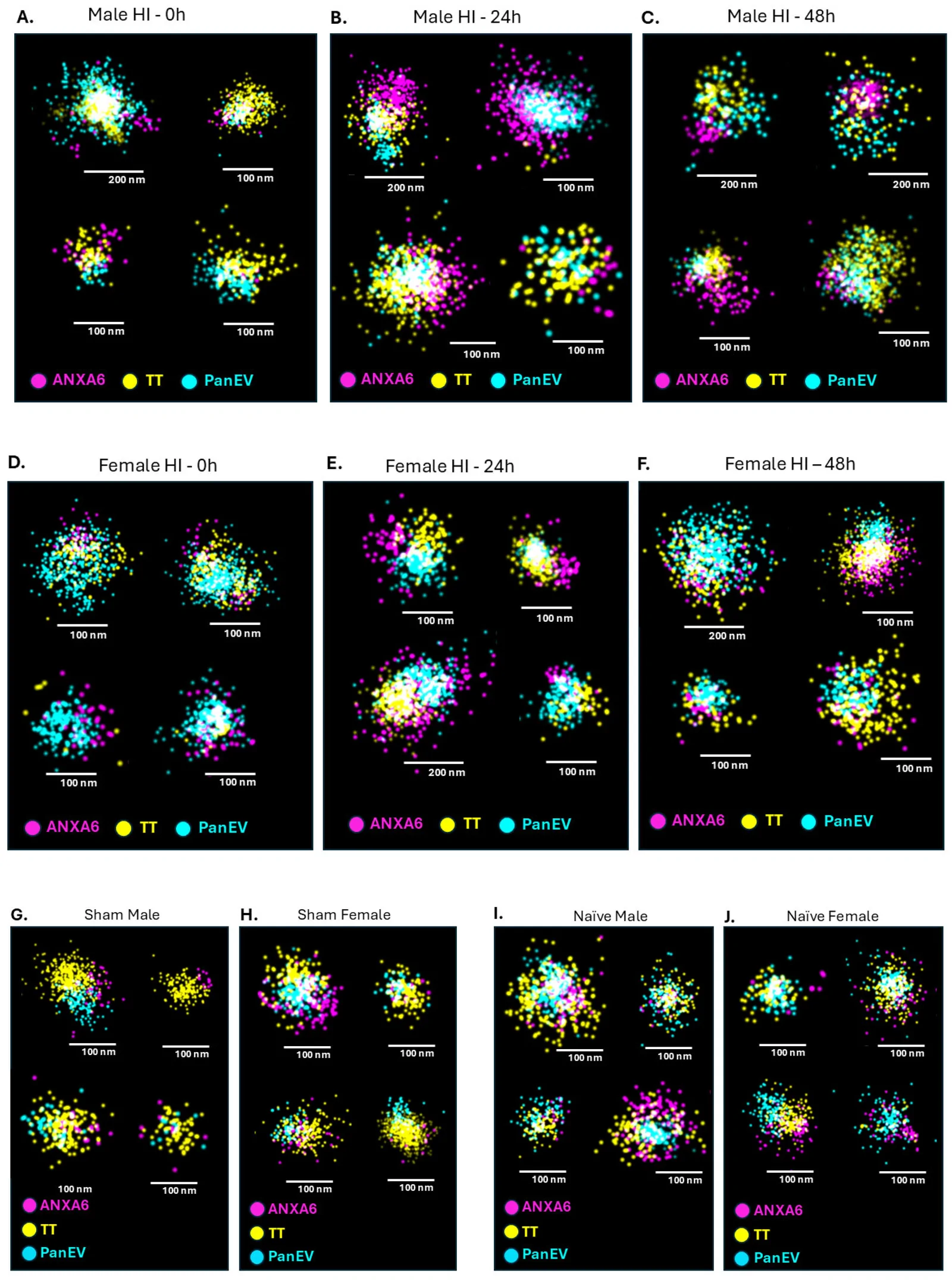
Examples of dSTORM imaging of ANXA6-positive staining co-localised to EVs labelled with PanEV and tetraspanin trio (TT) staining from all experimental groups. (**A**–**C)** EVs from male HI animals at 0 h (**A**), 24 h (**B**) and 48 h (**C**) post HI; (**D**–**F**) EVs from female HI animals at 0 h (**D**), 24 h (**E**) and 48 h (**F**) post HI; **G**,**H**) EVs from male (**G**) and female (**H**) sham controls at 0 h; (**I**,**J**) EVs from naïve male (**I**) and female (**J**) animals at 0 h. Scale bars are included in all figures, representing either 100 nm or 200 nm. ANXA6-positive labelling is shown in magenta, TT labelling in yellow and PanEV labelling in blue.

Detection of positive staining for ANXA6 protein EV cargo was identified in eye-lavage EVs of all experimental groups (30 min HI at 0 h, 24 h and 48 h post HI, sham and naïve animals at all time points) at differing % of total EVs, with examples of individually imaged EVs by dSTORM provided in Figure 4, showing co-localisation of ANXA6 to EV subtypes for all experimental HI groups (Figure 4A–C for males at 0 h, 24 h and 48 h post HI; Figure 4D–F for females at 0 h, 24 h at 48 h post HI) and examples from control groups (Figure 4G,H for male and female sham controls at 0 h and Figure 4I,J for male and female naïve animals at 0 h).

**Figure 5:**
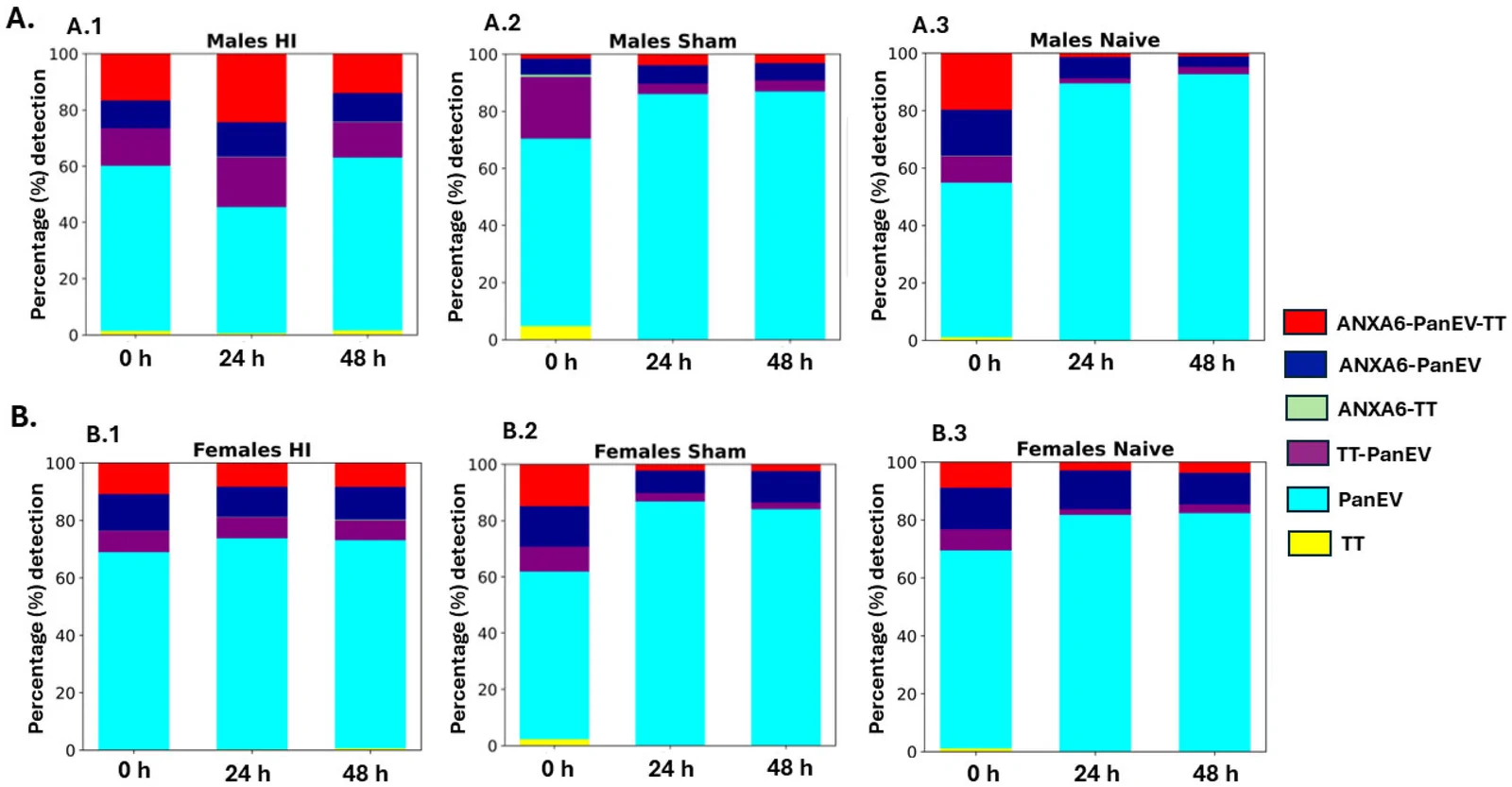
Column graphs showing proportional (percentage %) positive staining of eye-lavage EVs for EV subtype markers and ANXA6 EV cargo protein in all groups. (**A**) Male groups, eye-lavage EVs from HI (**A.1**), sham (**A.2**), and naïve (**A.3**) animals at 0 h, 24 h and 48 h time points. (**B**) Female groups, eye-lavage EVs from HI (**B.1**), sham (**B.2**) and naïve (**B.3**) groups at 0 h, 24 h and 48 h time points. Raw percentages per count are presented in Supplementary Table S1A and B.

The positive detection of ANXA6 labelling and EV markers (TT and PanEV) was explored for all experimental groups, including with respect to sex (male, female) at 0 h, 24 h and 48 h post mild (30 min) HI. Eye lavage from naïve and sham male and female control groups was also included for all three time points (Figure 5). When analysing the three time points of the HI model, in males there was the highest ANXA6-positive detection (37% positivity of total EVs) at 24 h post HI, compared with 26% and 24% of EVs staining positive for ANXA6 at 0 h and 48 h post HI, respectively (Figure 5A.1). Most of the ANXA6-positive signal was detected in the PanEV-TT co-stained EVs (red column), followed by the PanEV single-stained EVs (dark blue column), while only a very small proportion of ANXA6 staining was co-localised to the TT single-positive EVs (green column), with 1% detected in the 24 h group (Figure 5A.1). In comparison, the sham male groups showed notably lower ANXA6 staining for the EVs from all three time points (8%, 10% and 3% for 0 h, 24 h and 48 h, respectively) compared with the HI groups (Figure 5A.2). The naïve males showed higher ANXA6 staining (35%) of EVs at the 0 h time point, similarly to the HI groups, but notably lower levels at the 24 h (9%) and 48 h (5%) time points (Figure 5A.3), emphasising the high ANXA6 levels observed for the male HI groups post injury, particularly at the 24 h but also the 48 h time points post injury (Figure 5A.2). In the females, less ANXA6-positive staining was observed than in the males in all three HI groups, with 23%, 19% and 20% positivity in the 0 h, 24 h and 48 h post-HI time points respectively (Figure 5B.1). ANXA6-TT-positive EVs were detected at similarly low concentrations in the three HI groups (2%; Figure 5B.1). In the female sham groups, ANXA6 levels were higher in the 0 h time point group (29%) compared with the 24 h (10%) and 48 h (13%) time points, where ANXA6 levels were observed as reduced (Figure 5B.2). In the naïve female groups, ANXA6 detection was observed as reduced from what was observed between the 0 h (23%) and 24 h (16%) and 48 h (14%) time points (Figure 5B.3). This indicates developmental-related differences in ANXA6 EV cargoes, overall reducing within the 48 h time window for the developmental controls for both sexes, while detection was higher in the HI groups, particularly in the males (more notable at 24 h than 48 h post HI).

Furthermore, there were some differences observed in the distribution of the EV specific markers in the EVs not co-labelled with ANXA6; both between sexes and experimental groups. In all three HI groups, the TT-PanEV-positive EVs (purple column) were at a higher proportion in the males (13%, 18% and 13% for 0 h, 24 h and 48 h, respectively) (Figure 5A.1) than in the females (8%, 7% and 7% for 0 h, 24 h and 48 h, respectively) (Figure 5B.1). Differences observed between the sexes in the sham group were notably higher in the males at 0 h but otherwise similar between the sexes (9%, 3%, 2% in females and 21%, 4%, 4% in males at 0 h, 24 h and 48 h, respectively) (Figure 5A.2,B.2). Levels of TT-PanEV-positive EVs were similar between the sexes in the naïve groups (7%, 2%, 3% in females and 9%, 2%, 3% in males at 0 h, 24 h and 48 h, respectively (Figure 5A.3,B.3). The proportion of PanEV single-labelled EVs (light blue column) was overall lower in the HI male groups (59%, 45% and 61% for 0 h, 24 h and 48 h, respectively) (Figure 5A.1) compared with the HI female groups (69%, 74% and 73% for 0 h, 24 h and 48 h, respectively) (Figure 5B.1). Similar levels of PanEV staining were detected across time points in the sham male (66%, 86% and 87% for 0 h, 24 h and 48 h, respectively) and sham female (60%, 87% and 84% for 0 h, 24 h and 48 h, respectively) groups (Figure 5A.2,B.2), indicating increases in PanEV staining from 0 h to 48 h. In the naïve animals, PanEV staining also showed an increase in both sexes from the 0 h to 48 h time points comparing males (54%, 89% and 93% for 0 h, 24 h and 48 h, respectively) and females (68%, 82% and 82% for 0 h, 24 h and 48 h, respectively) (Figure 5A.3,B.3). This may be indicative of some developmentally related dynamics of EV subpopulations. TT single-labelled EVs were at negligible levels across all groups (Figure 5A,B).

**Figure 6:**
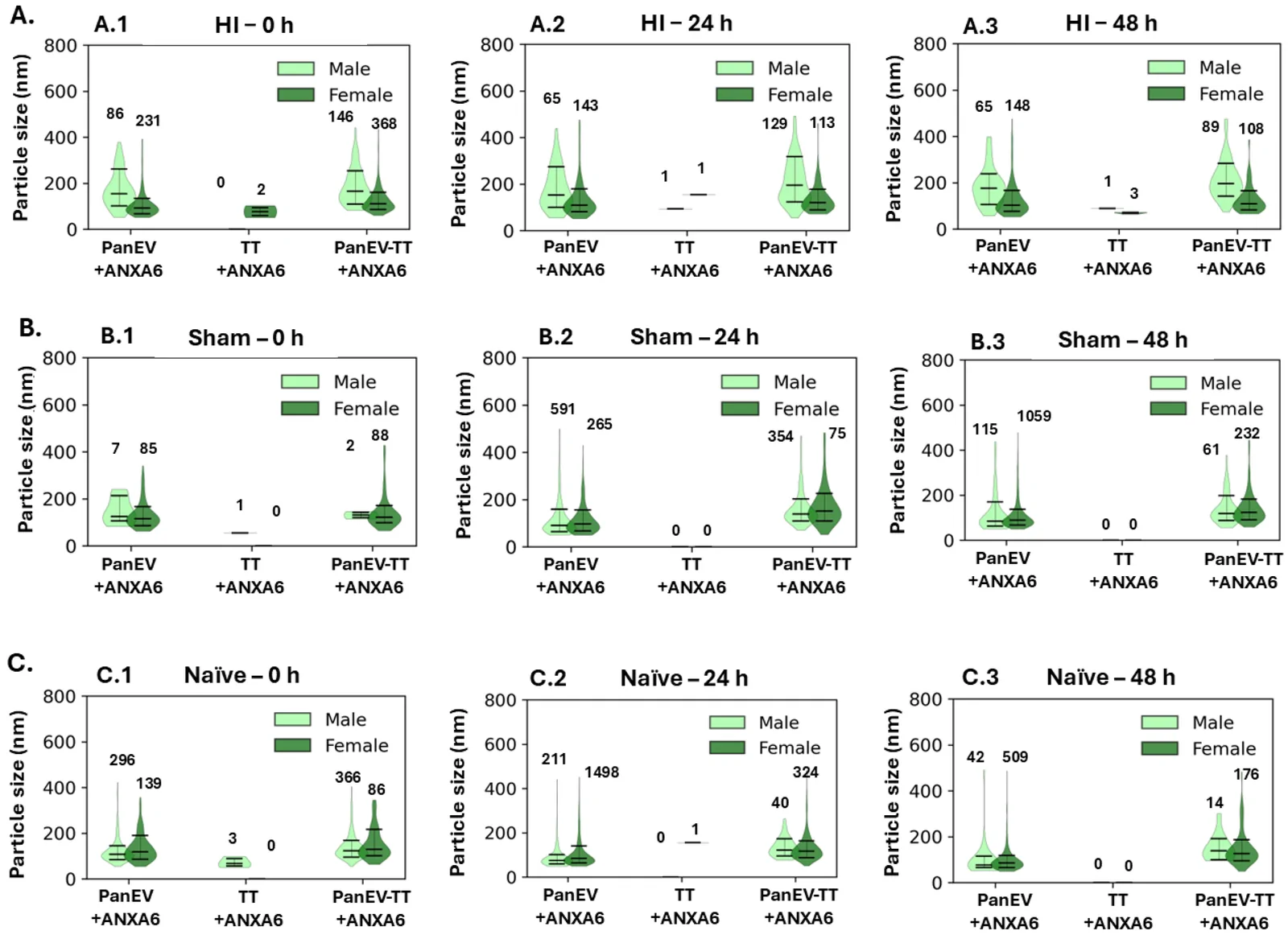
Size profiles of ANXA6-positive eye-lavage EVs in males and females at different time points post HI, as well as naïve and sham controls, presented as violin plots. (**A**) ANXA6-positive EVs at 0 h post HI (**A.1**), at 24 h post HI (**B.1**), and at 48 h post HI (**C.1**). (**B**) ANXA6-positive EVs in sham controls at 0 h (**B.1**), 24 h (**B.2**) and 48 h (**B.3**). (**G**–**I**). (**C**) ANXA6-positive EVs in naïve animals at 0 h (**C.1**), 24 h (**C.2**), 48 h (**C.3**). Median size with standard deviation is shown in all violin plots; counts of EVs behind each plot are indicated per group; EV analysis was filtered for particle size 50–500 nm.

In addition, all ANXA6-positive EVs were further analysed for size profiles per co-staining, with respect to ANXA6-PanEV-, ANXA6-TT- and ANXA6-PanEV-TT-positive EVs. Figure 6 shows violin plots of EV size distributions as estimated by dSTORM detection using the EV2Profile software in Codi (particle size was filtered for a 50–500 nm range). ANXA6-positive eye-lavage EVs are shown from males and females at 0 h (Figure 6A.1), 24 h (Figure 6A.2) and 48 h (Figure 6A.3) post HI, as well as from naïve (Figure 6B (B.1–B.3)) and sham (Figure 6C (C.1–C.3) animals. In all groups, PanEV single-positive EVs co-localising with ANXA6 staining were identified mainly in the 50–300 nm size range with a somewhat larger range in the males, approximately 50–450 nm, in all HI groups (Figure 6A.1–A.3) as well as the sham groups (Figure 6B.1–B.3), while in the naïve group the size profiles were overall smaller (approximately 50–300 nm; Figure 6C.1–C.3). For the TT single-positive EVs co-localising with ANXA6 staining, at the 0 h time point post HI these were only detected in females and not in males (Figure 6A.1). They showed a slightly wider range in the females at 24 h post HI (up to 180 nm compared to 150 nm in the males; Figure 6A.2), while the 48 h groups post HI showed very similar distribution between the sexes (Figure 6A.3), and the same was observed for the sham groups at the 0 h time point (Figure 6B.1). In the naïve 0 h group the TT+ANXA6-positive EVs in the males were up to 100 nm while in the females they were mainly in the 50 nm range (Figure 6C.1). When assessing the PanEV+TT+ co-labelled EVs also positive for ANXA6, a slightly wider size range was observed in the males (approximately 50–500 nm) compared with the females (approximately 50–300 nm) in the HI groups at all time points (Figure 6A.1–A.3), while profiles were similar between the sexes in the naïve 0 h group (Figure 6C.1) but slightly smaller in the male compared with the female 0 h sham group (Figure 6B.1).

## 3. Discussion

New biomarkers are urgently needed for assessment of HI damage, including for sex-associated differences, given more severe outcomes in males [21,28,29,30]. The biomarker potential of eye-lavage EVs to reflect brain damage post HI was recently highlighted by our group for mild and severe HI using a neonatal mouse model in mixed sex groups [8]. The current study used the same mouse model to assess sex-specific EV profiles, with a focus on ANXA6 as one of the specific EV cargo proteins previously identified at 48 h post mild HI in an experimental group of mixed sex [8]. The aim here was to identify whether ANXA6 could be an indicative EV-associated biomarker in mild neonatal HI, including with respect to sex and different time points (0 h, 24 h and 48 h) post HI insult. Using dSTORM imaging, ANXA6-positive labelling was quantified and co-localised to EV subtypes. In addition, the EV labelling of eye-lavage EVs was further interrogated with respect to EV markers in male and female mouse pups separately at different time points post HI.

Annexins are an evolutionarily conserved multigene family of proteins. They bind to negatively charged phospholipids in a Ca^2+^-dependent manner and have been recognised for their role in membrane repair, in addition to cell migration and adhesion [31]. ANXA6 has been implicated in many biological processes, including cell proliferation, survival, differentiation, inflammation, and also membrane repair.

Overall, in our current study, ANXA6-positive labelling was observed at higher levels in the male HI groups compared to their female counterparts, with a peak of ANXA6 positivity in the male group at the 24 h time point post HI (Figure 5). EVs were labelled with PanEV and tetraspanin trio (CD9, CD63 and CD81) markers. In the HI groups, ANXA6 co-labelled mainly with the PanEV+TT+ double-labelled EVs, followed by the PanEV+-labelled EVs, with lowest co-localisation with only TT+ in males. In the females, similar levels of ANXA6 positivity were observed in the PanEV+TT+ co-labelled EVs as in the PanEV+-only EVs. This indicates some differences in ANXA6 co-localisation to EV subtypes between the sexes following HI. PanEV+ANXA6 and PanEV+TT+ANXA6 EV populations in HI were furthermore observed to be larger in size on average (median difference) in males vs. females. This relationship was observed to be weaker in the naïve groups and was not observed in sham groups. This aligns with reports relating stress responses to increased EV size profiles [32,33] but remains observational at this stage. Due to the explorative nature of the current datasets, based on pooled samples from 10 eyes for each group, further in-depth statistical analysis was not considered appropriate and will remain subject to future studies on repeated biological samples in larger sample cohorts.

Deregulations in annexin expression and activity have been correlated with human diseases, e.g., in acute promyelocytic leukaemia and the antiphospholipid antibody syndrome, and the term annexinopathies has been created [34]. ANXA6 can influence intracellular Ca^2+^ homeostasis by interacting with calcium channels and transporters, making it an integral part of cellular signalling and communication. The role of ANXA6 has been mainly studied in muscle membrane repair and as a scaffold protein. Similarly to muscle, neuronal membrane damage can occur from physical trauma, from degenerative processes, or as a secondary consequence of a primary disease. In primary neurons genomically encoded ANXA6 was expressed at levels sufficient to form a repair cap at the site of injury [31]. ANXA6 has been shown to have protective roles in neuronal membrane dynamics [24] and in response to neuronal injury [23]. Roles for ANXA6 in neuroinflammation and synaptic plasticity in HI and cerebral ischaemia have been reported in previous studies [27,35]. In neonatal HI the Na+/K+ pumps stop functioning and accumulation of Na^+^, Ca^2+^, and Cl^-^ within the cell occurs. This Ca^2+^ overload causes neuronal membrane depolarisation and activation of lipases, proteases, and endonucleases, leading to destruction of the cellular skeleton [1,36]. As ANXA6 is a Ca-dependent binding protein, an increase in Ca^2+^ would likely increase ANXA6 levels as observed in our study. The observed increase in ANXA6 could also suggest higher injury levels with ANXA6 possibly contributing to the repair. Our findings may therefore be of considerable interest, as they point to some sex-specific differences following neonatal HI, not reported to date, as well as possible roles for EV-mediated transport of ANXA6 in response to brain injury.

Moreover, EVs carry a diverse set of bioactive molecules that can promote cell adhesion and motility and within those ANXA6 has been also identified [37]. Autophagy is a major type of cell death in neonatal HI, as well as in CNS development [1]. EVs facilitate autophagy while several annexins, including ANXA6, belong to the top 100 proteins often identified in EVs [37]. Therefore, possible developmental decreases in autophagy and respectively of ANXA6, as observed in our naïve groups (Figure 5), could be expected but will need to be further investigated in future studies.

As males are reported to be more susceptible than females to long-term cognitive deficits following neonatal HI, it is hypothesised that the sexual dimorphism of HI is due to the dimorphism in cell death taking place in males compared to females, i.e., autophagy vs. mitophagy respectively [38]. We observed the same pattern of ANXA6-positive eye-lavage EVs in sham and naïve male and female animal groups at all three time points, suggesting developmental reduction of ANXA6, and respectively we hypothesise a possible link to autophagy. Furthermore, it can be postulated that the increased levels of ANXA6 in the male HI eye-lavage EVs at 24 h and 48 h post HI observed here could be indicative of increased levels of autophagy due to the sustained injury compared to sham and naïve male controls at the same time points, while the female groups are less affected as a result of the mitophagy type of clearing of damage and debris. Therefore, it may be suggested that an increase in ANXA6 expression in the eye-lavage EVs in the male groups following HI could be indicative of elevated autophagy levels, but this will require further investigation.

Interestingly, ANXA6 is considered to mediate calcium-dependent EV secretion during plasma membrane repair [39]. In cancer models, ANXA6 was shown to affect proinflammatory cytokine secretion and associated EV release [25]. Furthermore, ANXA6 exported via EVs from cancer-associated fibroblasts has been linked to cancer drug resistance [40], while chemotherapy-elicited EVs were identified to be enriched in ANXA6 [41]. Roles for EV-exported ANXA6 remain poorly understood in relation to brain injury and hence our current findings may be of value for understanding ANXA6-mediated functions during different time windows post HI, as well as possible sex-related differences.

Furthermore, when assessing the EV subtype labelling of eye-lavage EVs for PanEV+, TT+ and PanEV+TT+ respectively in this study, some differences were observed between males and females, alongside deviations between time points post HI. When comparing time windows within each sex group the PanEV+-only-labelled EV levels were observed to be proportionally higher in the females compared with the males in all three HI groups, as well as in the sham groups at 48 h and naïve groups at 24 and 48 h. PanEV+TT+ co-labelled EV levels were observed to be proportionally higher in females than males at the 0 h post HI time point, as well as in the 24 h and 48 h female naïve groups. TT+ staining was overall comparatively low in all groups but not detected in the 24 h sham females and 24 h naïve males.

Overall, our findings indicate differences in dynamics of EV subpopulations released in eye lavage in response to neonatal HI in this mild HI mouse model, including with respect to sex and time post injury (0 to 48 h), as well as in response to developmental changes between P9 and P11. To our knowledge, this is the first assessment of such EV marker composition by dSTORM in eye-lavage EVs and in any other biofluid EVs in relation to HI. In stroke, dSTORM imaging of cytoskeletal protein has been reported as a useful method for identifying mechanisms underlying stroke tissue damage [42]. Single-EV detection by Raman spectroscopy was also recently described as a neuroinflammatory marker in traumatic brain injury [35]. The use of single-molecule detection of EVs and EV-associated cargo markers by dSTORM, reported in our current study, will contribute to furthering current understanding of cell-mediated mechanisms in responses to neonatal HI brain injury and aid development of diagnostic approaches and neuroprotection.

It must be noted that the EVs were captured by PS binding and, therefore, there may be some bias towards positive labelling with the PanEV marker compared with the TT marker. As our previous optimisation with the ONI kit showed PS binding to be more successful in capturing EVs from mouse fluids than TT binding when using the antibodies provided by ONI with the EV2 profiler kit [8], this approach was also applied in our current study. Given that the antibodies for the two EV surface markers supplied with the ONI kit are optimised for human samples it may be possible that further optimisation for application in mouse models may be needed. In addition, translatability from findings in the HI mouse model used in this study must next be tested in human clinical samples of eye lavage from neonates. Due to the small eye size of mouse pups at this stage of development, the current study utilised pools of n = 10 eyes per group for isolation of sufficient eye-lavage EVs for all the analyses carried out in this study, with 10 individuals used for the HI groups and five individuals for the control groups. These limitations need to be considered regarding stringent assessment for identifying individual variability in our current pilot study and therefore some caution should be applied when interpreting the data. We did though observe some indications of proportional differences between sexes and experimental/control groups, highlighting the potential for ANXA6 as a sex-specific male biomarker associated with mild HI. When using the neonatal mouse HI model, to achieve the concentration needed for EV labelling quantification, the eye lavage obtained from 10 eyes had to be concentrated to 50 μL in total, for application on the ONI chips, NTA, blotting and TEM. As this is an explorative proof-of-concept study, assessment in additional pools of n = 10 for further biological replicates of our reported results is outside the scope of the current evaluation and does not apply to the 3Rs. It must furthermore be highlighted that the study focusses on the ipsilateral eye to assess the effects of combined hypoxia and ischaemia (using the ipsilateral eye from 10 animals per sex), compared with sham and naïve controls (using both eyes from five animals per sex for each control group as we did not anticipate differences between the two eyes as the animals were not subjected to hypoxia, ischaemia or both). The contralateral eye, and its respective brain hemisphere, in the HI group was subjected to only hypoxia and was not used, as the ipsilateral side in the Rice–Vannucci model mimics the HI insult in humans and is therefore of direct clinical relevance.

EV-based therapeutics are promising in brain injury diagnostics and treatment [11,43,44,45], yet the development of standardised techniques, including in neonatal scenarios, remains a challenge [46]. Compared with EVs from plasma or serum, the use of eye-lavage EVs offers a non-invasive approach and possibly a clearer link to brain injury due to the eye–brain connection [7]. Previous studies in a neonatal HI mouse model reported putative HI-specific plasma-EV markers while sex differences were not interrogated [47]. Neuronal proteins have been identified as possible biomarkers in serum EVs of TH-treated near-term/term neonates following HI [48]. However, both these studies [47,48] offer an invasive approach. Moreover, biomarkers detected in serum might not be the same for plasma, and the results may be inconsistent. It therefore remains essential to identify candidate robust and sex-specific biomarkers for HI which have a potential to be associated with either mild or severe HI. This will help in developing therapies tailored for male and female neuroprotection post HI [49].

## 4. Materials and Methods

### 4.1. Mild HI Mouse Model

Mild neonatal HI brain injury was carried out using the modified Rice–Vannucci neonatal mouse model, with unilateral carotid artery ligation, followed by exposure to 10% oxygen for 30 min. The mild HI brain injury model is confirmed and detected histologically, using well-established markers, including microglial activation, cell death and tissue loss post mortem at 48 h post injury, as well as behavioural and histological assessments at later time points [8,43,50,51].

**Figure 7:**
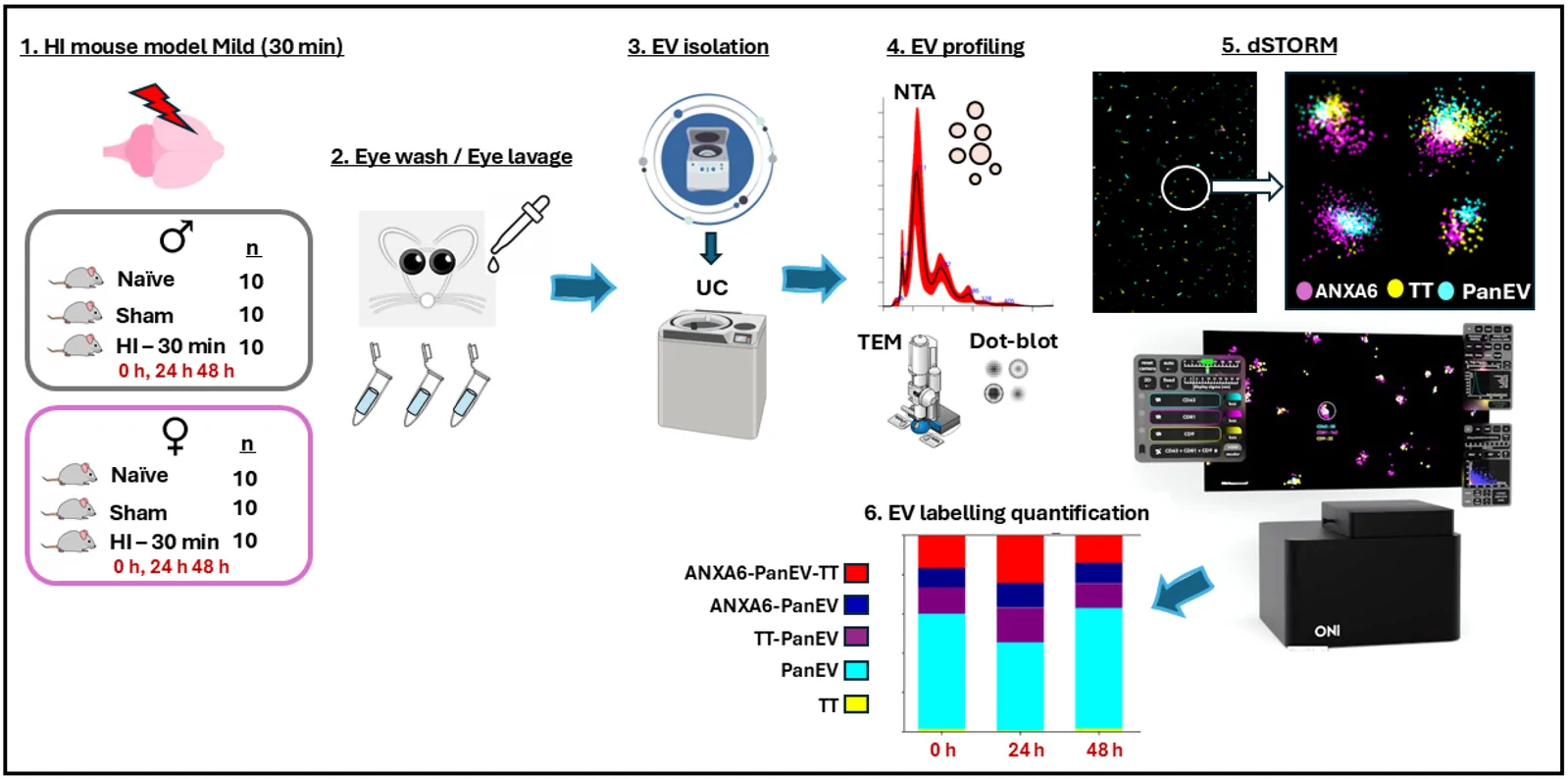
Experimental setup and workflow. The mild HI model was performed on male and female mouse pups, including naïve and sham controls. Eye lavage was collected and pooled from 10 eyes per experimental group, due to the small eye surface size to obtain sufficient EV concentration in downstream analysis. EV isolation was performed by established protocols using differential centrifugation. EV profiling was conducted using nanoparticle tracking analysis (NTA), transmission electron microscopy (TEM) and dot blotting of EV surface markers. The EVs were then labelled and visualised by single-molecule detection using super-resolution microscopy by dSTORM (using the EV Profiler 2 kit, ONI). EV labelling was quantified using Bayesian framework analysis. ANXA6-positive staining of EVs was analysed in all groups and co-localisation with EV specific markers (PanEV, TT) determined.

All experiments on animals and associated care protocols were performed in accordance with UK Home Office approval (PP0028535), the UCL Animal Welfare and Ethical Review Board, and the United Kingdom Animals (Scientific Procedures) Act 1986. The ARRIVE guidelines were followed. Operations were performed on C57/BI6 mice (Charles River, United Kingdom), which were bred in house, at post-natal day 9 (P9), using an adaption of the Rice–Vannucci model of neonatal HI as previously described [8,43,50,51,52]. The parental animals were bred in an environment providing a 12 h light/dark cycle and food and water ad libitum. The HI procedure for mild (30 min HI) was carried out as follows: P9 mice (males and females) were anesthetised using isoflurane (5% induction, 1.5% maintenance). Permanent occlusion of the left common carotid artery was established with 8/0 polypropylene sutures followed by wound closure with tissue glue. The pups were recovered at 36 °C and then were returned to the dam for 2 h, whereafter they were placed in a 36 °C hypoxic chamber for 30 min (mild HI) in humidified 10% oxygen/90% nitrogen at 3 L/min. The pups were returned to the dam and eye lavage was collected at 0 h, 24 h and 48 h for EV isolation. The study used n = 10 eyes per group, per sex, separating the ipsilateral and contralateral eye, to use the eye lavage from the ipsilateral side in this study, as this is the clinically relevant eye for the HI groups. Naïve animals served as controls (n = 5 animals per time point, per sex, using both eyes per animal, n = 10 eyes per group). In addition, sham-operated animals were exposed to incision and wound closure but not carotid artery occlusion or hypoxia (n = 5 animals per sex per time point, using both eyes per animal, n = 10 eyes per group). We did not anticipate hypoxia- or ischaemia-related changes in the control groups, as they were not exposed to these conditions, and we did not anticipate signature differences in relation to ANXA6 or EV markers between baseline left and right eyes, hence both eyes were used from each animal. The total number of animals used in this study was therefore 120 (60 males and 60 females). Animals from all litters collected for this study were separated into males and females and randomly allocated for experimental and control (sham and naïve) groups. All litter pups were included, and no animals were subjected to exclusion. Only the researcher extracting the samples was aware of the group allocation and the analyses were done blindly, without decoding of groups until the raw data was collected. A summary of the experimental setup and workflow is provided in Figure 7.

### 4.2. Eye-Lavage EV Isolation

Eye-lavage EVs were collected according to previously described methods [8], washing the pups’ eyes with DPBS (20 µL per eye). For the HI animals, eye lavage was collected separately from both eyes of 10 animals per group, for assessment of the left eye (affected by HI) for the current study. For the naïve and sham groups, eye lavage was collected and pooled from both eyes from 5 animals (n = 10 eyes per group). For all groups, a pool of eye lavage from 10 eyes was therefore used for EV analysis per group.

Eye-lavage EVs were isolated by differential centrifugation and ultracentrifugation as previously described [8]. In brief, eye lavage was first centrifuged at 4000 g for 20 min to separate apoptotic bodies and aggregates. The supernatants were collected into fresh Eppendorf tubes, centrifuged at 30,000 g for 1 h at 4 °C, collecting the EV-enriched pellet and discarding the supernatant. The EVs were washed in 500 µL DPBS by briefly vortexing the EV pellet and spinning it again at 30,000 g for 1 h at 4 °C. The final EV pellet (from a pool of 10 eyes per group) was reconstituted in 50 µL DPBS before further analysis.

EVs were imaged using transmission electron microscopy (TEM) as previously described [8,53], fixed by glutaraldehyde, stained by uranyl acetate and imaged using a JEOL JEM 1400 microscope (Tokyo, Japan) and digital images captured using a GATAN Rio16 digital camera (Ametek GB Limited, Leicester, UK). Dot blotting was used to detect CD63 and Flotillin-1, two established EV surface markers, according to methods described in Li et al., 2025 [8]. In brief, 10 µL EV solution was dotted onto nitrocellulose membranes using a 10 µL pipette and allowed to dry for 20 min. The membranes were blocked in 5% BSA in TBS-T before primary antibody incubation (1/1000 in TBS-T) for CD63 (ab216130, Abcam, UK) and Flotillin-1 (ab41927) overnight at 4 °C. Visualisation was carried out using antirabbit IgG HRP-conjugated antibody (BioRad, Watford, UK) in conjunction with ECL (Amersham, Merck, UK) and the UVP BioDoc-ITTM System (Cambridge, UK) for digital capture.

Nanoparticle tracking analysis (NTA) using the NS300 Nanosight (Malvern Panalytical, Ltd., Malvern UK) was used to assess EV numbers and size distribution profiles of the EV pools (n = 10) for each group. EVs were diluted 1/100 in DPBS (10 µL EV solution in 990 µL DPBS) and applied for NTA with an automated syringe pump (set at speed 50), with five 60 s readings recorded and averaged per sample pool. For capture, camera level was set at 11 and for post-analysis the threshold was set at 5. Replicate histograms from the recordings were generated by the NTA software (version 3.0; Malvern Panalytical), showing EV median count, modal size and mean size with standard error.

### 4.3. Direct Stochastic Optical Reconstruction Microscopy (dSTORM) for Super-Resolution Imaging of ANXA6 EV Cargo Detection and Co-Localisation to EV Markers

Single-molecule localisation-based super-resolution microscopy imaging of the eye-lavage EVs was carried out to visualise the ANXA6 EV cargo protein of interest and EV surface markers (PanEV and tetraspanin trio (anti-CD9 + CD63 + CD81), TT), all of which were labelled with fluorescently tagged antibodies. The antibody for ANXA6 (Anti-Annexin 6/ANXA6 antibody, ab196942, Abcam, Cambridge, UK; reported to cross-react with mouse [54]) was conjugated to AlexaFluor-647 in house (STFC, RAL, Harwell, UK) to use it for co-labelling experiments in conjunction with the ONI EV Profiler 2 kit (Oxford, UK). Visualisation of the EVs by dSTORM was carried out on an ONI Nanoimager system (ONI UK, Oxford, UK). Samples were prepared following the manual sample preparation protocol (EV Profiler 2 kit, ONI). Briefly, phosphatidylserine (PS) was used to capture EVs on the chip surface and chips were prepared for permeabilised EVs for intraluminal EV cargo detection according to the manufacturer’s instructions, applying the exact same protocol and time durations for the different incubations to all sample preparations on all chips for the different sample groups. Chips were prepared in parallel, with all parameters set the same for all lanes and chips. EV volume applied for each lane was 10 μL, with EV concentration at approximately 1 × 10^10^/mL, according to the kit’s specifications. EV visualisation was carried out by labelling with tetraspanin trio marker (anti-CD9 + CD63 + CD81 (561)) and a PanEV marker (488), with ANXA6 as the cargo protein of interest (labelled with Alexa-647 fluorophore) and co-localised to EV subtypes. Freshly prepared dSTORM imaging buffer was applied to each chip before immediately imaging using the Nanoimager S Mark II system with the AutoEV imaging and CODI (https://alto.codi.bio/) analysis protocol according to the manufacturer’s specifications (ONI). Laser settings were as follows for imaging: 180 mW of 488 nM laser, 44 mW of 561 laser and 170 mW of 640 nM laser. Imaging was carried out at 40 ms/frame with 3000 frames for the 488 nm laser and 1000 frames for the 561 and 640 nm lasers, using a minimum of six fields of view for each sample lane per run, to stay within the duration of the 90 min span of the dSTORM buffer being active for each run. Each chip was re-imaged with fresh dSTORM buffer applied for the second imaging to gather information from a total of 12 fields of view per sample group. A boundary of at least 100 µm between ROIs in the first and second reads was furthermore used to avoid the risk of cross-ROI photobleaching or possible double-counting. Initial data reports were generated by AutoEV, reporting the proportion of labelled and co-labelled EVs from each sample group. For quantification of labelled EVs, datasets (EV positivity) were recovered using the following particle filters: diameter 50–500 nm, label counts > 5 for the two EV characterisation markers (PanEV and TT) as these form part of the EV structure, and > 2 for the ANXA6 cargo protein marker, according to the manufacturer’s recommendations for EV cargo detection using the EV Profiler 2 kit (ONI). Analysis of co-staining was thereafter carried out using previously developed and described techniques based on a Bayesian framework [53].

### 4.4. Statistical Analysis

GraphPad Prism (version 10) and matplotlib (version 3.7.2) in Python (version 3.10) were used for comparison of datasets from the different study groups. Given the explorative nature of this pilot study, based on a pool of n = 10 eyes per experimental group, any in-depth statistical comparison was omitted as biological replicate-level variability could not be estimated. Therefore, the data assessment remains descriptive, and the results should be interpreted as exploratory comparisons of pooled proportions rather than definitive estimates of biological variability.

## 5. Conclusions

This explorative pilot study identified some sex-related differences in eye-lavage EV compositions, indicating that an increase in ANXA6-positive EVs may be associated with early changes in response to mild HI, particularly in males. It is of great importance to identify candidate sex-specific biomarkers for HI, particularly given the worse neurological outcomes in male babies suffering HI insult. It must be noted that the current study was carried out in a mouse model of mild neonatal HI, and hence some caution is needed for direct translation to human babies, with further validation required including in large translational animal models for paediatric studies, such as piglets, as well as in clinical human neonatal samples. Findings of this study will contribute to a pipeline for future development of non-invasive EV-based biomarkers from eye lavage with clinical value for HI.

## Data Availability

The original contributions presented in this study are included in the article/Supplementary Materials. Further inquiries can be directed to the corresponding author.

## Supplementary Materials

The following supporting information can be downloaded at: https://www.mdpi.com/article/10.3390/ijms27198875/s1.
