# Supplementary material for: Annexin A6 in Eye-Lavage Extracellular Vesicles—a Candidate Biomarker for Sex-Specific Differences in Mild Hypoxic–Ischaemic Brain Damage—Findings from an Explorative Neonatal Mouse Model Study
Source: Int J Mol Sci. 2026 Oct 5;27(19):8875. doi: 10.3390/ijms27198875 (PMC13665257; doi:10.3390/ijms27198875)
Supplement: Supplementary file 1 [file ijms-27-08875-s001.zip › ijms-4540886-supplementary.pdf]

Supplementary Figure S1.

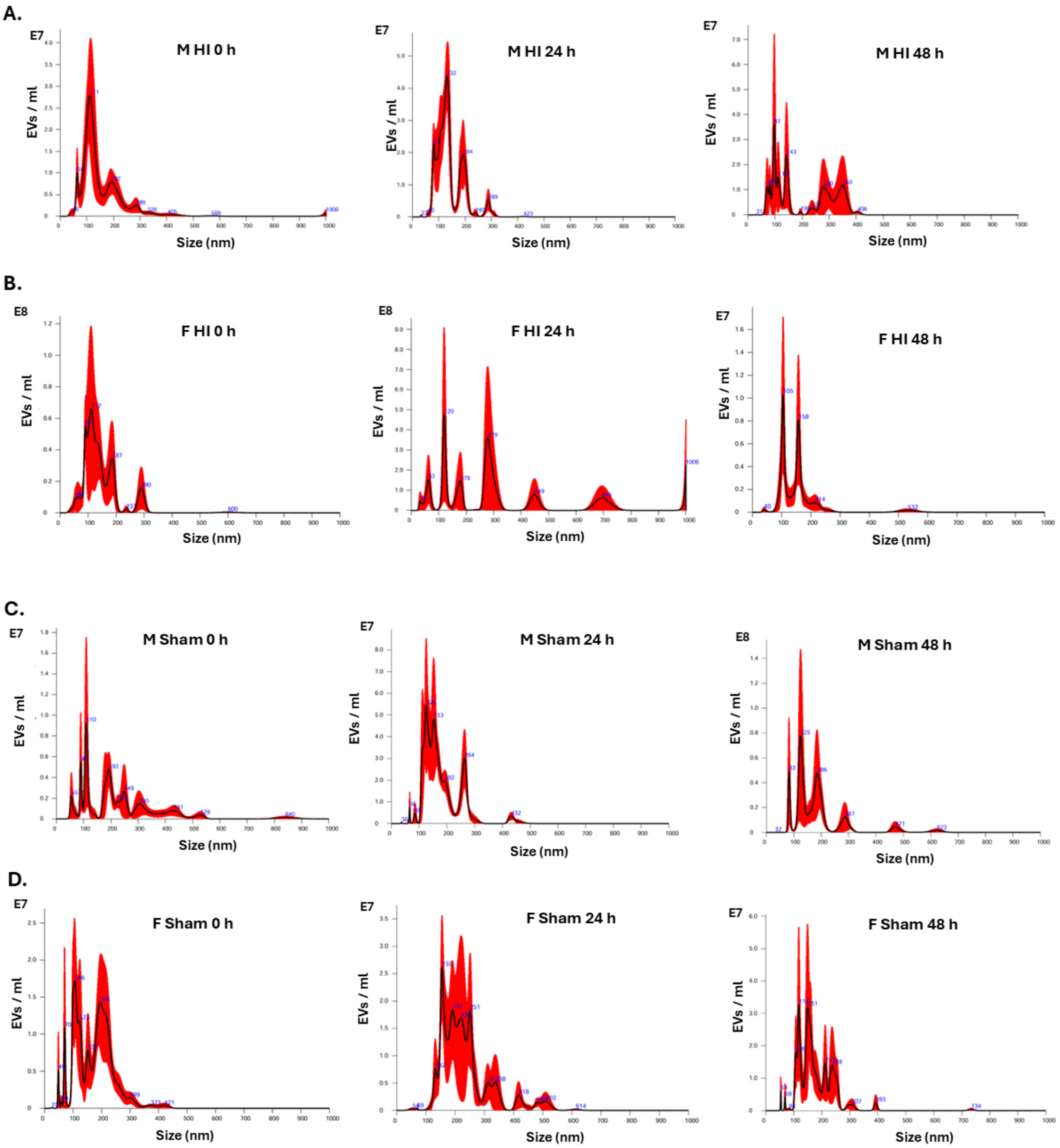

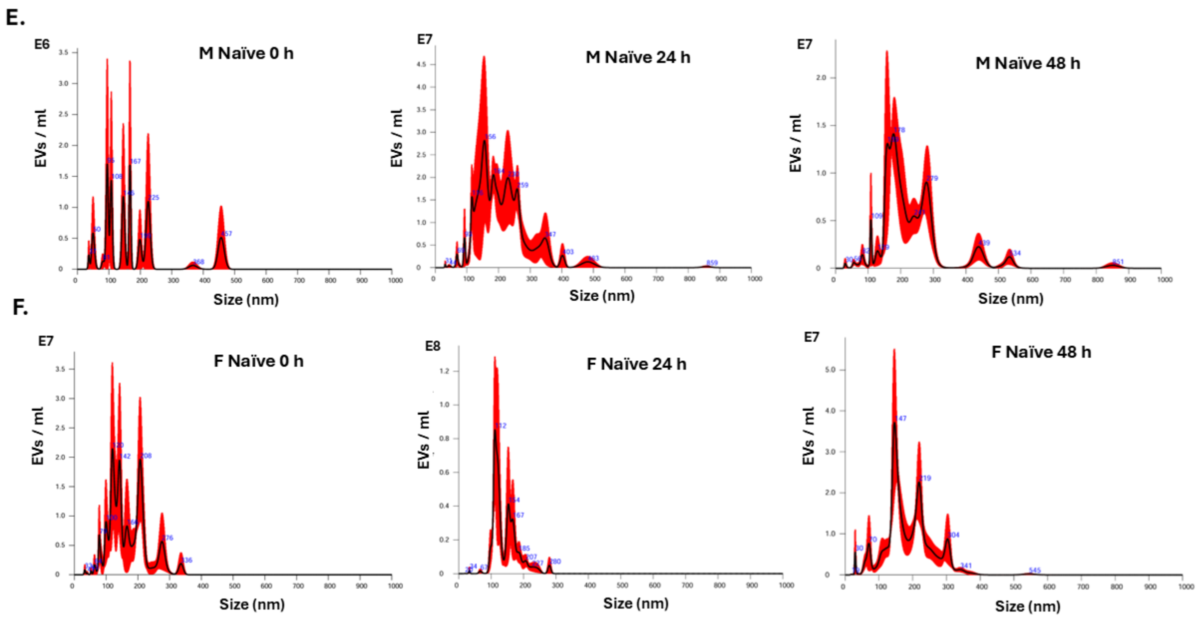

Supplementary Figure S1. NTA graphs of eye-lavage EVs from all experimental groups. **A)** Males at 0 h, 24 h, 48 h post HI; **B)** Females at 0 h, 24 h, 48 h post HI; **C)** Male shams at 0 h, 24 h and 48 h; **D)** Female shams at 0h, 24 h, 48 h; **E)** Male naïve animals at 0 h, 24 h, 48 h; **F)** Female naïve animals at 0 h, 24 h, 48 h. The black line represents the mean and the red line represents the standard error for the mean (SEM) of each NTA graph, which shows the EV size distribution for each group composed of a pool of eye-lavage collected from n=10 eyes.

Supplementary Table S1. Raw percentages for EV positive staining behind column graphs in Figure 5, detecting TT, PanEV and ANXA6 staining.

A. Male Groups – HI, Sham, Naïve.

		**HI**	**Sham**	**Naive**
**Time point**	**EV staining category**	**Percentage**	**Percentage**	**Percentage**
**0 h**	TT	1.37	4.8	1.08
**0 h**	PanEV	58.74	65.6	53.83
**0 h**	TT_PanEV	13.37	21.6	9.22
**0 h**	ANXA6_TT	0	0.8	0.16
**0 h**	ANXA6_PanEV	9.83	5.6	15.97
**0 h**	ANXA6_TT_PanEV	16.69	1.6	19.74
**24 h**	TT	0.57	0.03	0
**24 h**	PanEV	44.91	86.02	89.47
**24 h**	TT_PanEV	17.74	3.61	1.75
**24 h**	ANXA6_TT	0.19	0	0
**24 h**	ANXA6_PanEV	12.26	6.47	7.38
**24 h**	ANXA6_TT_PanEV	24.34	3.87	1.4
**48 h**	TT	1.57	0.1	0.09
**48 h**	PanEV	61.48	86.81	92.55
**48 h**	TT_PanEV	12.58	3.91	2.57
**48 h**	ANXA6_TT	0.16	0	0

**48 h**	ANXA6_PanEV	10.22	6	3.6
**48 h**	ANXA6_TT_PanEV	13.99	3.18	1.2

B. Female Groups, HI, Sham, Naïve.

		**HI**	**Sham**	**Naïve**
**Time point**	**EV staining category**	**Percentage**	**Percentage**	**Percentage**
**0 h**	TT	0.18	2.2	1.13
**0 h**	PanEV	68.75	59.66	68.35
**0 h**	TT_PanEV	7.48	8.81	7.32
**0 h**	ANXA6_TT	0.06	0	0
**0 h**	ANXA6_PanEV	12.7	14.41	14.33
**0 h**	ANXA6_TT_PanEV	10.84	14.92	8.87
**24 h**	TT	0.22	0	0.02
**24 h**	PanEV	73.53	86.77	81.74
**24 h**	TT_PanEV	7.4	3.01	1.96
**24 h**	ANXA6_TT	0.07	0	0.01
**24 h**	ANXA6_PanEV	10.48	7.97	13.38
**24 h**	ANXA6_TT_PanEV	8.28	2.26	2.89
**48 h**	TT	0.62	0.01	0.02
**48 h**	PanEV	72.5	84.02	82.35
**48 h**	TT_PanEV	6.93	2.39	3.04
**48 h**	ANXA6_TT	0.23	0	0
**48 h**	ANXA6_PanEV	11.4	11.14	10.83
**48 h**	ANXA6_TT_PanEV	8.32	2.44	3.75
